# Diagnostic Approach to Lower Limb Entrapment Neuropathies: A Narrative Literature Review

**DOI:** 10.3390/diagnostics13213385

**Published:** 2023-11-04

**Authors:** Nicu Cătălin Drăghici, Vitalie Văcăraș, Roxana Bolchis, Atamyrat Bashimov, Diana Maria Domnița, Silvina Iluț, Livia Livinț Popa, Tudor Dimitrie Lupescu, Dafin Fior Mureșanu

**Affiliations:** 1 “IMOGEN” Institute, Centre of Advanced Research Studies, 400012 Cluj-Napoca, Romania; nicu.draghici@umfcluj.ro; 2 “RoNeuro” Institute for Neurological Research and Diagnostic, 400364 Cluj-Napoca, Romania; silvina.ilut@yahoo.com (S.I.); livia.popa@ssnn.ro (L.L.P.);; 3 Department of Clinical Neurosciences, “Iuliu Hațieganu” University of Medicine and Pharmacy, 400012 Cluj-Napoca, Romania; 4 Faculty of Medicine, “Iuliu Hațieganu” University of Medicine and Pharmacy, 400347 Cluj-Napoca, Romania; bolchis.roxana@yahoo.com (R.B.);

**Keywords:** entrapment neuropathies, nerve compression syndromes, peroneal neuropathy, fibular neuropathies, tibial neuropathy, sural nerve, sciatic neuropathy, deep gluteal syndrome, piriformis muscle syndrome, meralgia paresthetica

## Abstract

Entrapment neuropathies of the lower limb are a misunderstood and underdiagnosed group of disorders, characterized by pain and dysesthesia, muscular weakness, and specific provoking movements on physical examination. The most frequent of these syndromes encountered in clinical practice are fibular nerve entrapment, proximal tibial neuropathy, sural nerve neuropathy, deep gluteal syndrome or sciatic nerve entrapment, and lateral femoral cutaneous nerve entrapment, also known as meralgia paresthetica. These are commonly mistaken for lumbar plexopathies, radiculopathies, and musculotendinous diseases, which appear even more frequently and have overlapping clinical presentations. A comprehensive anamnesis, physical examination, and electrodiagnostic studies should help clarify the diagnosis. If the diagnosis is still unclear or a secondary cause of entrapment is suspected, magnetic resonance neurography, MRI, or ultrasonography should be conducted to clarify the etiology, rule out other diseases, and confirm the diagnosis. The aim of this narrative review was to help clinicians gain familiarity with this disease, with an increase in diagnostic confidence, leading to early diagnosis of nerve damage and prevention of muscle atrophy. We reviewed the epidemiology, anatomy, pathophysiology, etiology, clinical presentation, and EDX technique and interpretation of the entrapment neuropathies of the lower limb, using articles published from 1970 to 2022 included in the Pubmed, MEDLINE, Cochrane Library, Google Scholar, EMBASE, Web of Science, and Scopus databases.

## 1. Introduction

Entrapment neuropathies of the lower limb are an often misunderstood and overlooked group of conditions, often leading to chronic pain and other disabilities if not treated in a timely manner. Among the most frequent of these syndromes encountered in clinical practice are fibular nerve entrapment, proximal tibial neuropathy, tarsal tunnel syndrome, sural nerve neuropathy, deep gluteal syndrome or sciatic nerve entrapment, and lateral femoral cutaneous nerve entrapment, also known as meralgia paresthetica. 

Moreover, within these compression neuropathies, several gradings of peripheral nerve injury changes, such as neuropraxis, which causes conduction blocks, or axonotmesis, which involves axonal damage, have been described in detail by Seddon and Sunderland [1]. Also, injury to the peripheral nervous system initiates a sequence of molecular changes in the nerve segment. Thus, at the pathophysiology level, in entrapment neuropathies, a cascade of events can occur such as: Schwann cell demyelination, proliferation and re-myelination, or Wallerian degeneration and axonal growth [2,3]. Additionally, a series of surgical and non-surgical therapies, including pharmacological, electrical, cell-based, and laser therapies, have been employed to promote myelination and enhance functional recovery after peripheral nerve injury [4].

Furthermore, this narrative review aims to provide a clearer overview of these common entrapment neuropathies by reviewing the anatomy, pathophysiology, clinical presentation, and Electrodiagnostic (EDX) findings in patients with these pathologies. By focusing on these prevalent syndromes, this article intends to bridge the gap in understanding, leading to more effective diagnosis, management, and improved patient outcomes. A comprehensive literature search was conducted using the databases from Pubmed, MEDLINE, Cochrane Library, Google Scholar, EMBASE, Web of Science, and Scopus. The search was limited to articles published between 1970 and 2022. Subject-specific keywords tailored to each respective entrapment syndrome, such as “Entrapment Neuropathy”, “Compression Neuropathy,” and “Electrodiagnostic,” were employed in combinations, facilitated by Boolean logical operators (AND, OR, NOT). Searches were also performed using Medical Subject Headings (MeSH) terms, where applicable. Language restriction to English and Romanian were applied.

## 2. Fibular Nerve Entrapment (Peroneal Nerve Entrapment)

### 2.1. Introduction

Fibular or peroneal nerve entrapment neuropathy is the most common mononeuropathy of the lower extremity and the third most common after median and ulnar neuropathies [5,6,7]. The most frequently affected site is at the fibular head, where the nerve’s superficial trajectory predisposes it to injury [8,9].

### 2.2. Anatomy, Etiology, and Pathophysiology

**Figure 1:**
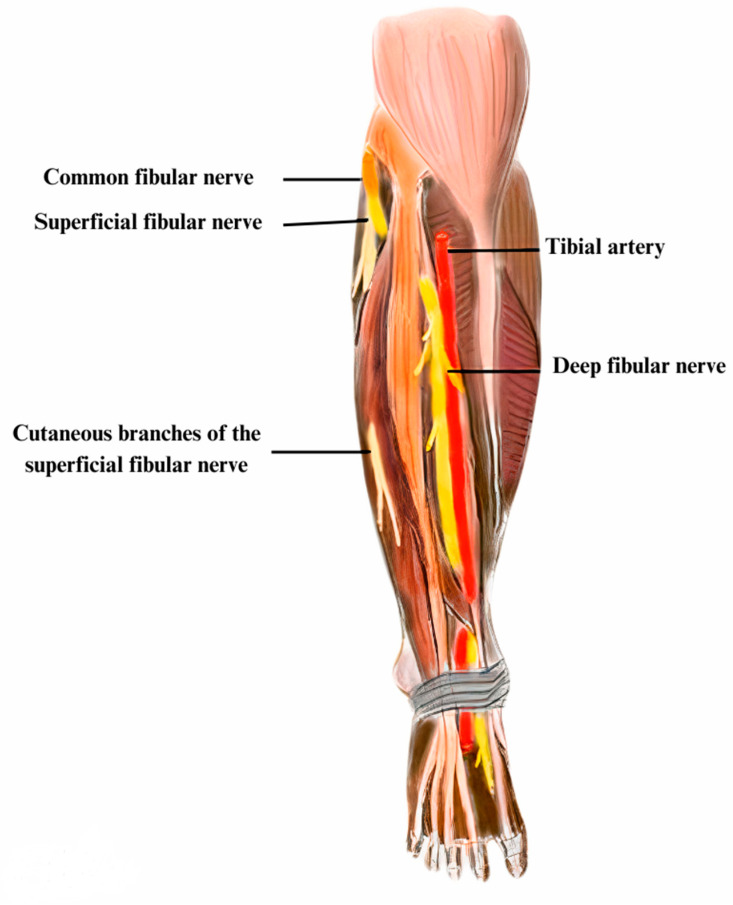
The common fibular nerve, originating from the sciatic nerve near the knee, bifurcates at the fibular head into the deep fibular nerve and the superficial fibular nerve, providing innervation to the lateral compartment muscles responsible for foot eversion.

**Figure 2:**
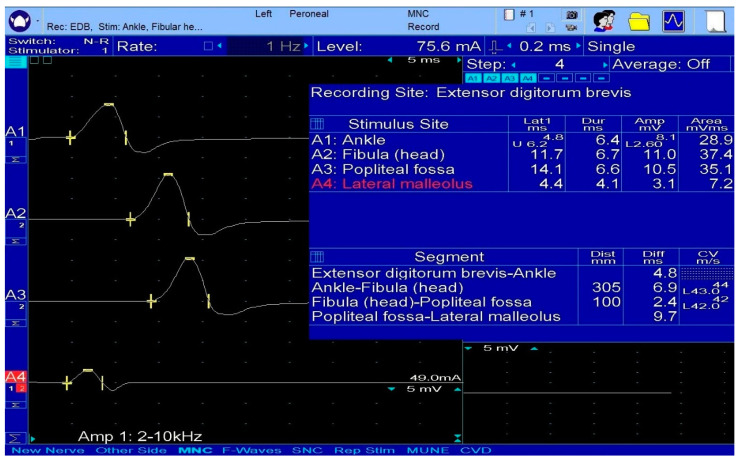
Fibular nerve with low CMAP at the supramaximal bimalleolar stimulation, compared to the CMAP amplitude of the nerve, below the fibular head. An accessory fibular nerve is suspected, which can be detected by applying an electrical stimulus behind the lateral malleolus.

The common fibular nerve (fibular nerve or external sciatic nerve) branches off from the lateral division of the sciatic nerve [10]. At the superior popliteal fossa, it splits into the superficial fibular nerve and the deep fibular nerve [5,7,11] (Figure 1). The superficial fibular nerve’s terminal divisions are the medial dorsal and the intermediate cutaneous nerves [5,7]. A common anatomical variant is represented by the accessory fibular nerve, present in approximately one-quarter of the general population, which supplies the innervation of the extensor digitorum brevis muscle [7] (Figure 2).

For the common fibular nerve, the most typical site for compression is at the fibular head [11,12]. Other compression sites are: (1) the exit of the lateral leg compartment, as it pierces through the crural fascia, for the superficial fibular nerve; and (2) in the tight tunnel formed by the extensor retinaculum muscle above, and the navicular and talus bones underneath, for the deep fibular nerve [5].

Acute presentations are usually related to high energy trauma, such as knee dislocations, fibular fractures, and ankle trauma, while chronic presentations are a result of behavioral causes or masses, such as ganglion cysts and neuromas [5]. Moreover, knee arthroplasty represents a risk of fibular nerve entrapment [6] as well as prolonged immobilization [11]. Diabetes is also a predisposing condition due to the edema caused by sorbitol in the nerve tissue [13]. Likewise, behavioral causes can precipitate a neuropathy through a repetitive cross-legged position, prolonged periods of squatting, and extrinsic compression due to bed rest [7,11]. Weight loss may also induce fibular neuropathy, associated with a secondary loss of subcutaneous tissue and, therefore, a greater risk of compression [11]. 

### 2.3. Clinical Presentation and Physical Examination

Symptoms occur acutely or insidiously, depending on the precipitating cause [5,7]. Fibular neuropathy is the most frequently reported lower extremity mononeuropathy in athletes [7], dancers [5], or occupations requiring squatting or kneeling for an extended period of time [8].

**Figure 3:**
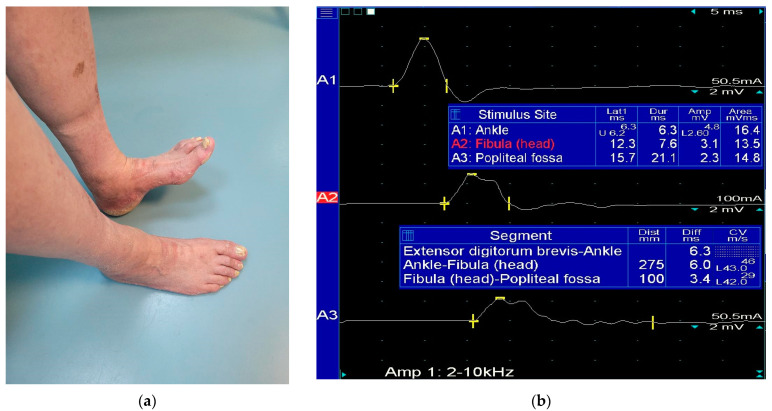
(**a**) Weak ankle dorsiflexion at the level of the right leg; (**b**) ENG examination confirm a conduction bloc at the level of the fibular head and decrease VCM at this site.

A typical patient suffering from fibular nerve entrapment presents with foot drop (Figure 3), resulting in steppage gait, pain, and numbness of the lateral lower leg and foot dorsum [5,11], which are symptoms typically aggravated by plantar flexion and foot inversion [6]. 

Common fibular nerve entrapment neuropathy presents with weakness of foot eversion and dorsiflexion of the ankle, weakness of great toe extension and sensory loss, and burning, tingling, and pain in the anterolateral distal leg and foot dorsum [5]. 

Superficial fibular nerve entrapment neuropathy symptoms are rarely present in isolation and are most commonly exacerbated during exercise [6]. Weakness of foot eversion is similar to the previous entrapment neuropathy, but ankle dorsiflexion and great toe extension are normal. The sensory abnormalities are the same as with common fibular nerve entrapment neuropathy, present at the foot dorsum and lateral site of the shin, with sparing of the dorsal space of the first web space and fifth toe [5]. It occurs more commonly in soccer players [7].

Deep fibular nerve entrapment neuropathy or anterior tarsal tunnel syndrome give few sensory symptoms, or the patient could be asymptomatic [5]. Numbness and paresthesias in the first web space that awaken the patient from sleep [7], together with pain or dull aching in the anterior ankle and dorsal foot that worsens with tight shoe wearing, could be present [5]. 

The differential diagnosis of foot drop with proximal neuropathies such as sciatic mononeuropathy, lumbosacral plexopathy, and lumbar radiculopathy is vital. In addition to the signs and symptoms of fibular neuropathy described above, these pathologies present with foot inversion and plantar flexion weakness; medial, lateral, and plantar foot sensory loss; and a reduced Achilles reflex. The L4-S1 lumbar radiculopathies also present with hip abduction weakness [8,11]. For the definitive diagnosis, however, electrodiagnostic studies are needed. 

### 2.4. Electrodiagnostic and Imaging Techniques

To differentiate between common fibular nerve, superficial fibular nerve, and deep fibular nerve entrapment neuropathy and other neuropathies, EDX studies are the definitive assessment tool. EDX studies are also necessary for injury severity, specifically to differentiate between an axonal or demyelinating injury, thus guiding prognosis for potential nerve function recovery [5,9,11]. Therefore, the presence of compound muscle action potential (CMAP) in the affected territory is associated with a good prognosis [7]. As well, contralateral comparison is useful for quantifying the severity of axonal loss [8].

For the common fibular nerve evaluation, motor nerve conduction studies (NCSs) are carried out with the G1 recording electrode placed on the midpoint of the extensor digitorum brevis muscle and the G2 electrode in the fifth metatarsophalangeal joint [14]. The stimulating electrode has three separate sites: at ankle level, laterally to the tibialis anterior tendon, and above and 10 cm below the fibular head [7,14]. A stimulation sweep speed of 5 ms/division and gain at 5 mV/div are used. The frequency filter is set between the low 2 Hz and high 10 kHz [14]. The conduction block can be identified when the decrease in CMAP amplitude is less than 50% [11].

EMG should be conducted on the tibialis anterior, at least. Additionally, the peroneus longus and, less often, the short head of the biceps femuralis muscles can be studied [5]. The tibialis anterior muscle, innervated by the deep fibular branch, is the most likely to show abnormal findings (denervation activity, neurogenic motor unit action potentials (MUAP)) [7]. The short head of the biceps femoris is studied since it is the only fibular innervated muscle above the knee, thus it will help to localize the lesion above or below the knee [7,8]. 

Antidromic sensory NCS of the superficial fibular nerve’s branches is recommended [12]. The recording electrode for sensory response is placed at the inframalleolar line, over the intermediate and medial dorsal cutaneous nerves, or 3 cm proximally to the bimalleolar line. The stimulating electrode is placed at the anterior edge of the fibula, proximal to the recording electrode with a distance of 12–14 cm [7,15]. A stimulation sweep speed of 1 ms/division and gain of 20 microV are applied. The frequency filter was set between the low 30 Hz and high 2.000 Hz [15]. Moreover, in the case of unknown etiology, ultrasound could be useful in the visualization of scarring, bone infiltrations, and mass lesions, with contralateral comparison rendering the most accurate results [5,11]. Magnetic Resonance Imaging (MRI) can also be used [11]. It identifies T2 hyperintensity lesions in the nerve trajectory and denervation signs of the anterior and lateral muscle compartments. For the deep fibular nerve, it can also show fascial defects and muscle herniation, with axial imaging in dorsiflexion being recommended [10]. 

## 3. Proximal Tibial Neuropathy

### 3.1. Introduction 

Proximal tibial neuropathy (PTN) is defined as entrapment of the tibial nerve in the popliteal fossa by impinging masses or by the fibrous sling of the soleus muscle. The latter is also known as soleal sling syndrome [16,17]. 

### 3.2. Anatomy, Etiology, and Pathophysiology

The sciatic nerve branches into the common fibular nerve and the tibial nerve at the distal thigh level. The tibial branch continues its tract through the popliteal fossa and passes over the popliteal muscle. In order to enter into the deep posterior compartment of leg muscles, it passes under the tendinous arch of the soleus muscle. Additionally, along its tract, the nerve is accompanied by the tibial artery and vein [16]. Due to its deep location, the nerve is usually not affected by external traumatic accidents. The main etiological reason for PTN entrapments is space-occupying lesions. We found several cases in the literature: tibial arterial aneurysm [18] and pseudoaneurysm [16], intraneural ganglion cysts [19], Baker’s cysts [20], tibial bone exostosis [21], and popliteal muscle enlargement [22]. In these cases, the space-occupying lesions either shifted the nerve to the fibrous sling of the soleus muscle or were the primary reason for entrapment, by compressing the nerve with mass effect. Although space-occupying lesions are more common, primary nerve entrapment by the fibrous sling of the soleus muscle can be the reason for PTN [17]. 

### 3.3. Clinical Presentation and Physical Examination

The dominant symptoms are pain and weakness in the popliteal, calf, and plantar muscles. These can become worse with active plantar flexion or passive dorsiflexion of the foot and ankle [17]. Moderate weakness of toe flexion or muscles can also be present. The dominant sensory symptoms are numbness, paresthesia, hypersensitivity, and tingling in the sole and heel of the foot. Physical examination plays a huge role in the diagnosis and can be easily assessed. Many patients will have a positive Tinel’s sign, evoked by gently pressing the muscle tissue 8–9 cm below the tibial plateau, which can provoke a severe pain, radiating to the medial sole of the foot. In more advanced cases, muscle atrophies in the calf and plantar region can be seen as well [16,17,23]. 

Additionally, the tibial nerve can be compressed distally along its path. Thus, tarsal tunnel syndrome (TTS), is an entrapment neuropathy of the posterior tibial nerve or one of its branches that is associated with the compression of these structures in the tarsal tunnel. The incidence of TTS is unknown, but up to 43% of patients have a history of trauma including events such as ankle sprains [24]. The clinical diagnosis is based on a detailed history and clinical examination, but electrophysiological studies and imaging techniques, such as plain X-rays, ultrasounds, or MRI, provide additional information [25]. Several characteristic signs and symptoms of TTS include poorly localised paraesthesia, dysesthesia, and hyperaesthesia radiating from the retro-malleolar region to either the sole, heel, or digits of the forefoot, or a combination of these areas. Symptoms are typically unilateral and usually worsen with the progression of the day. Dependent on the pathologic aetiology, patients may display a distinct localized tenderness, mass, or swelling over the medial malleolar region [26]. Moreover, in patients with diabetic neuropathy, the tibial nerve tends to be larger compared with healthy controls, according to a recent meta-analysis, which could lead to higher chance of nerve entrapment as it travels through the narrow behind the malleolus [27].

### 3.4. Electrodiagnostic and Imaging Techniques

The deep location of the tibial nerve makes EDX evaluation very challenging. Therefore, many physicians rely on physical examination for diagnosis, and MRI and/or ultrasonography (US) for positive diagnosis. 

**Figure 4:**
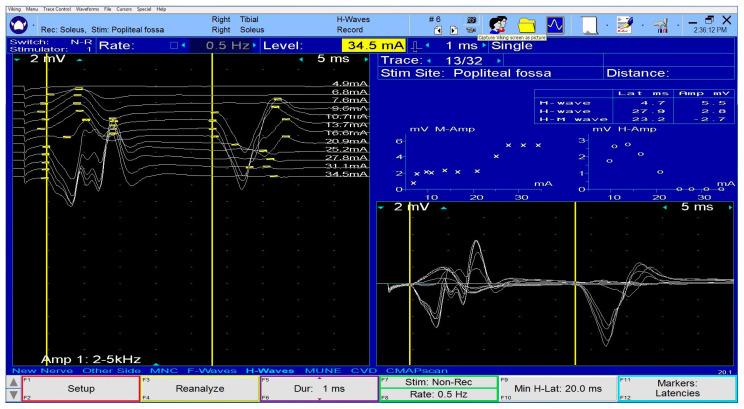
H-reflex with normal latency and amplitude at the level of the soleus muscle.

Even so, E. Fournier and D.C. Preston et al. described the recommended procedure of tibial motor study. The distal stimulation site is located at the ankle just behind the internal malleolus. A distal motor latency <5.5 ms and a CMAP amplitude >6 mV are normal values. The proximal stimulation site is located in the popliteal fossa, straddling the crease of the knee, on the axial line, or slightly outside it. The expected response is plantar flexion of the foot. The expected popliteal-ankle VCM is usually >42 m/s. The recording site is the abductor hallucis brevis muscle. The G1 electrode is placed at an equal distance of 1 cm proximal and inferior to the navicular prominence while G2 is placed over the metatarsal-phalangeal joint of the hallux [28,29]. The tibial CMAP is monitored and often high-intensity stimulations are required when stimulated at the popliteal fossa to ensure supramaximal stimulation. Moreover, the soleus H-reflex study can be helpful in PTN diagnosis. This reflex is absent or delayed in PTN. The stimulation site is identical with the proximal tibial motor study protocol, with the cathode being placed rostrally. The G1 recording site is 2–4 cm distal to the locus where the soleus meets the two bellies of the gastrocnemius, and the G2 recording site is just over the Achilles tendon. The H-reflex (Figure 4) occurs at low-intensity stimulations without a direct muscle response [28].

High Resolution UltraSound (HRUS) is a good tool to investigate the presence or absence of space-occupying lesions. Moreover, we can have the dynamic image of structures surrounding the nerve and help to differentially diagnose arterial claudication [30]. On the other hand, MRI can show proximal T2 nerve hyperintensity, which indicates venous congestion and blockage of normal axoplasmic flow. In addition, distally, we can have T2 hyperintensity due to Wallerian degeneration. As well, at the site of entrapment, the nerve will show abnormal flattening. Moreover, MRI can indirectly indicate neural entrapment by showing regional pathological changes associated with muscle denervation. In some cases, it was even possible to confirm the thickening of the fibrous sling of the soleus muscle [31,32,33].

## 4. Sural Nerve Neuropathy

### 4.1. Introduction

Because of its superficial location and extensive usage as a nerve graft, the sural nerve is one of the most well-studied nerves in human body. Moreover, it is one of the nerves with numerous anatomical variations; therefore, any type of compression of the nerve along its long trajectory will lead to sural nerve neuropathy (SNN) [34].

### 4.2. Anatomy, Etiology, and Pathophysiology

The sural nerve is derived from S1 and S2 nerve roots. In the most common anatomical variation, the sural nerve arises from two branches of the tibial nerve and common fibular nerve. The medial sural cutaneous branch emerges from the tibial nerve at the level of the distal popliteal fossa. It traverses the two gastrocnemius heads and is usually joined at mid-calf level by the communicating branch of the fibular nerve and forms the proper sural nerve. The trajectory of the nerve travels down the posterolateral side of the foot and lateral to the Achilles tendon, accompanied by the small saphenous vein (SSV). At the ankle, it passes to the lateral malleolus and posterior to the fibular tendons and bifurcates into the lateral calcaneal nerve and lateral dorsal digital nerve of the V finger [34,35,36]. 

The etiology of SNN damage can be distributed in three groups: (a) traumatic; (b) atraumatic; and (c) iatrogenic. Therefore, due to its superficial tract and close relationship with bone structures, any traumatic impact has a high risk of causing SNN. Ankle sprains and fractures of the distal fibula, talus, calcaneus, and the base of the fifth metatarsal are the most common traumatic causes. Atraumatic causes are myositis ossificans in the gastrocnemius, fibular or Achilles tendon tendinosis, osteochondroma, or space-occupying lesions, such as a Baker’s cyst, local schwannoma, and neuroma or intraneural ganglia. Nevertheless, the main cause of SSN remains iatrogenic. The superficial location and anatomical variations make the nerve susceptible to direct intra-operational traumas. Likewise, the close relationship with the anatomical structures represent a risk of nerve injury during their manipulation, such as: SSV varices ablation, Achilles or fibular tendon repair, gastrocnemius recession surgery, or arthoscopies [35,37,38,39,40].

### 4.3. Clinical Presentation and Physical Examination

The sural nerve is a purely sensory nerve and provides sensation for the lateral part of the ankle, heel, and foot up to the base of the fifth metatarsal bone. Thus, patients with SSN will complain about paresthesias, tingling, radiating pain, or/and tenderness in the above-mentioned areas. The pain and discomfort are exacerbated by physical activity and during nighttime. The physical examination is often unremarkable, with normal reflexes and unaffected motor components. Tinel’s sign may be positive but is unreliable in patients with good physical condition. Physicians can perform provocative maneuvers such as inversion and plantar flexion of the foot, which is expected to be positive [34,35,36].

### 4.4. Electrodiagnostic and Imaging Techniques

**Figure 5:**
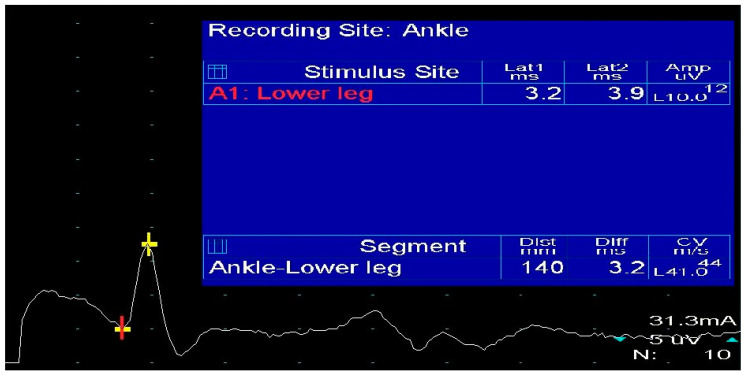
Orthodromic stimulation of the sural nerve at 14 cm from the active electrode point.

D.C. Preston and his team explain in detail the procedure of NCS of the sural nerve. The posterior-lateral calf represents the stimulation site and two points at the posterior ankle are the recording sites. G1 is located just posterior to the lateral malleolus while G2 is located 3–4 cm distal to G1. Low stimulation intensities (5–25 mA) and a duration of 0.1 ms are usually enough to achieve a supramaximal response (Figure 5). The optimal position for patients is lying on their side with the studied leg facing up. It is advised that the examination is performed on both feet and that the results are compared [28]. However, clinical presentation and anamnesis of the patient is usually enough to suspect potential SNN. The imaging studies are used to confirm the suspicion and to locate the exact site of entrapment and evaluate its severity. Ultrasound, or HRUS in particular, is often very efficient for this purpose. Plain radiographs are also useful, since fractures, osteochondromas, and myositis ossificans can also be the cause of SSN. MRI shows the presence of space-occupying lesions in soft tissues and localizes the entrapment site with high specificity [38,39,40].

## 5. Deep Gluteal Syndrome (Sciatic Nerve Entrapment)

### 5.1. Introduction

Deep Gluteal Syndrome (DGS) is characterized by the entrapment of the sciatic nerve (SN) in the subgluteal area. This complex includes several types of variants—piriformis syndrome (PS), gemelli-internal obturator complex syndrome, ischiofemoral impingement syndrome, and hamstring syndrome—and correlates with non-discogenic and extrapelvic etiologies having clinical presentations of pain and dysesthesias in the posterior region of the hip and buttocks [41,42]. PS is the most common explanation for DGS and is caused by sciatic nerve impingement by the piriformis muscle (PM) [43,44,45]. It is often overlooked and can vary from 6% to up to 17.8% of cases of chronic low back pain or sciatica [46,47,48]. The mean age of diagnosis is 43 years old, with a slight female predominance [47,49]. 

Gemelli-internal obturator complex syndrome involves the compression of the SN by the internal obturator muscle. The ischiofemoral impingement syndrome refers to the entrapment of the sciatic nerve by the quadratus femoris in the ischiofemoral space, whereas proximal hamstring syndrome refers to the impingement of the nerve by the semitendinosus, semimembranosus, and biceps femoris muscles, respectively [50].

### 5.2. Anatomy, Etiology, and Pathophysiology

**Figure 6:**
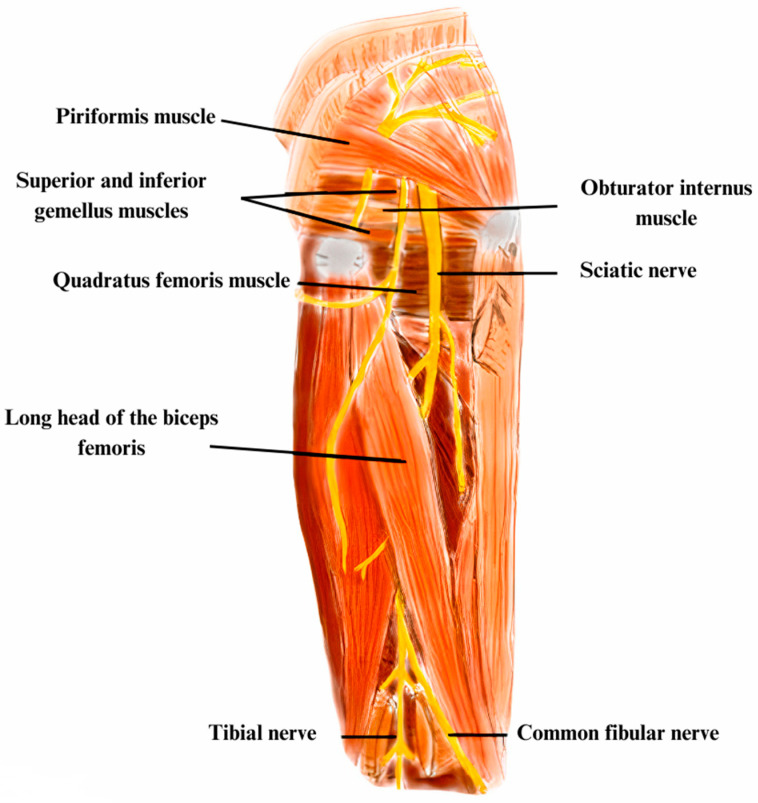
This is an illustration of the DGS, demonstrating the course of the sciatic nerve through the infrapiriformis compartment and its potential compression by the musculotendinous structures, primarily piriformis, and the adjacent gemelli-obturator internus complex. The anatomical juxtaposition of the proximal hamstring muscle group in relation to the sciatic nerve are further illustrated.

The SN derives from the L4-S3 ventral branch of the sacral plexus [45]. It can be trapped at the level of the lumbar spine, but also due to intrapelvic and extrapelvic pathologies in the subgluteal space or deep gluteal space. The subgluteal space is located anterior to the gluteus maximus muscle. It is positioned lateral to the tensor fasciae latae muscle and linea aspera, medial to the sacrotuberous ligament and inferior to the ischial tuberosity at the level of the proximal insertion of the hamstring muscles. The femur trochanters and neck are anterior [41,51]. The PM has the proximal insertion at the level of the anterolateral surface of the sacrum and the superior margin of the greater sciatic notch. Distal insertion is situated at the level of the superior greater trochanter (Figure 6). Its contraction determines the external rotation of the hip and, secondarily, abduction when it is flexed [48,52]. 

The trajectory of the SN nerve has several characteristics. After the passage out to the greater sciatic notch, the nerve lies down inferiorly to the PM, over the obturator internus muscle. Thus, this typical anatomical pattern causes a scissor effect with entrapment of the nervous structure [50,53]. At the thigh, it is situated posterior to the adductor magnus and anterior to the long head of the biceps femoris. Likewise, when it enters the popliteal fossa, it passes between the biceps and the semimembranosus muscles [45]

The etiologies that trigger DGS symptoms are varied. Pathologies which diminish SN mobility during joint movement—iatrogenic, traumatic, inflammatory, tumoral, or mechanical overuse etiologies—can cause nerve damage [50]. Moreover, several structures determine SN entrapment through acute edema: musculotendinous, osseous, neurovascular, or capsular tissue [45]. Vascular causes and endometriosis compress the SN as a result of the intimate relationship with the iliac vessels, ovaries, and sacral plexus [45]. Moreover, several congenital and acquired anomalies of the PM, internal obturator muscle, and sciatic nerves are reported. Nerve branches passing through one of the muscles, trajectory variants of the SN, and high variability in the insertions of the PM and accessory PM can cause SN entrapment [45,54]. Other causes include hypertrophy secondary to overuse, infection of the PM, and leg length discrepancy [43,47,55]. PM hematoma or fibrosis following trauma is a frequent etiology but, in some cases, the cause is unidentified [49]. In addition, several conditions such as strains, avulsions, and tendinopathies encountered in running and jumping sports predispose individuals to hamstring syndrome [44].

### 5.3. Clinical Presentation and Physical Examination

Some clinical features can explain the DGS manifestation: posterior thigh and hip area pain, and unilateral buttock discomfort with low back pain, sometimes with a radicular distribution, accompanied by tenderness [44,46,47,50]. These symptoms are exacerbated by walking and running, and hip flexion with knee extension—when the nerve is maximally stretched—and worsens at night [44,51]. The patient walks with a limp due to muscular weakness. At the same time, he cannot stand for more than 30 min and adopts an antalgic position, with the healthy ischium supporting the weight [41,45,51]. In patients with ischiofemoral impingement and hamstring syndrome, the pain is exacerbated when taking bigger steps or at the initial heel strike [44]. 

Furthermore, in several reviews, the PS is associated with a quartet of symptoms: (1) buttock pain—consistently present; (2) pain aggravation by sitting; (3) external tenderness near the greater sciatic notch; and (4) any PS provocative maneuver that determines PS symptoms [46,47]. Thus, some provocative maneuvers simulate the symptoms: the Lasègue test or straight leg raise; the Freiberg sign—in a supine position, active internal rotation of the hip; the Pace sign—resisted hip abduction; and the FAIR test—narrowing of the space between the PM and internal obturator muscle by flexion of the hip, adduction and internal rotation [50,51]. Additionally, Michel et al. devised a 12-point clinical assessment score for the diagnosis and treatment standardization of PS. The test’s sensitivity and specificity were 96.4% and 100%, respectively, for a score over 8 points, where the diagnosis was considered “Probable” [43]. Additionally, the PM muscle can also generate deep myofascial pain, exacerbated by prolonged walking or through squatting [49,55,56]. The palpation of the structures in the gluteal region can reveal a sensitive mass or tenderness to the IT, between the ischium and the femoral head. Thus, symptoms present (1) laterally and at the IT level, and are attributed to the hamstring syndrome and ischiofemoral impingement; and (2) medial to the IT, and are specific to forpudendal nerve entrapment. Moreover, the differential diagnosis of deep gluteal region pain with lumbosacral radiculopathies, sacroiliac or hip articular pathologies, and gynecological disease must be performed [44,45,57]. Thus, US- or CT-guided injections provide relief of symptoms and have diagnostic and prognostic, post-operative significance [45]. As well, several systematic reviews concluded that the diagnostic pathway must include anamnesis, physical examination, imaging (pelvic radiographs and MRIs), lidocaine or corticosteroid injection, and EDX findings [41,44]. 

### 5.4. Electrodiagnostic and Imaging Techniques

**Figure 7:**
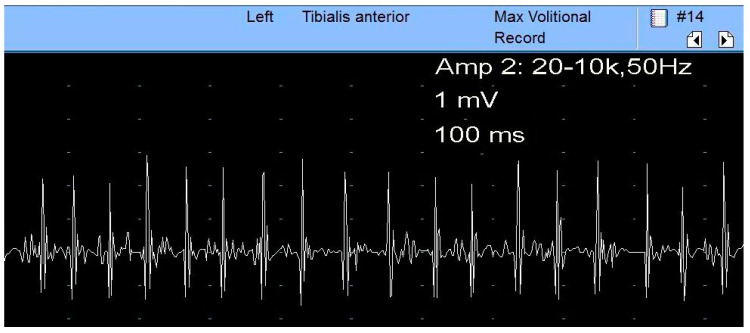
Chronic neurogenic process in the muscle biceps femoris highlighted by neurogenic MUPs with high amplitude and poor interference pattern.

For SN assessment, NCS and EMG should be bilaterally conducted. The NCS studies of the fibular and tibial nerve, respectively, the fibular superficial nerve and sural nerve, are described in their chapters. The F responses of the tibial and fibular nerves and the H-reflexes are usually prolonged and should be studied. The fibular nerve is more severely affected than the tibial nerve and, where there is axonal loss, the CMAP amplitudes are reduced. The EMG study narrows the lesion site and quantifies the severity of the disease. The study protocol involves the examination of two fibular and tibial innervated muscles, the long and short heads of the biceps femoris muscle, and one muscle innervated by the superior and inferior gluteal nerves [28]. Thus, if muscles innervated by the fibular and tibial nerves and the biceps femoris muscle present EMG reduced recruitment of MUAPs, an SN lesion can be confirmed (Figure 7). Moreover, if an anomaly is found in the muscles innervated by the superior and inferior gluteal nerves, a plexopathy or radiculopathy is more likely. However, the L5 and S1 paraspinal muscles must also be assessed for the differential diagnosis between radiculopathy and plexopathy [28,48,54]. 

Likewise, in the assessment of PS, the dynamic examination is mandatory, since NCS and EMG are frequently normal in the early stages of nerve injury [28]. Spontaneous EMG activity and an amplitude decrease in sensory and motor nerve action potential may appear after several weeks [48,54,58]. Therefore, at the onset of symptoms, the EDX studies may be normal and they must be repeated three to four weeks later [44]. At the same time, EDX studies exclude myopathies or neuropathies with overlapping symptoms, such as radiculopathies, sciatic nerve palsy, and hip joint-mediated pain [45,48,54,55].

US, CT, and MRI are used to exclude other pathologies, as well as for establishing the etiology. In addition, magnetic resonance neurography (MRN) can visualize PM and SN modifications [58]. Thereby, a study conducted by Filler et al. found MRN to have a specificity of 93% and sensitivity of 64% in diagnosing PS in patients with chronic sciatica and normal MRI results. PS was the major diagnosis established following MRN and interventional MRI imaging. The criteria consisted of asymmetry and SN hyperintensity [42]. Moreover, for DGS, MRI is mandatory, excluding discogenic causes of sciatica [41,44]. CT and US visualize hematomas, tumors, and abscesses which could cause buttock pain. Periarticular endoscopic examination of the subgluteal space can detect the cause of entrapment and simultaneously decompress the nerve [45,50,51,57]. 

## 6. Meralgia Paresthetica (Lateral Femoral Cutaneous Nerve Entrapment)

### 6.1. Introduction

Meralgia paresthetica (MP), also known as Bernhardt–Roth syndrome, is a purely sensory mononeuropathy which involves compression of the lateral femoral cutaneous nerve (LFC) at different sites along its trajectory [59,60,61,62]. This syndrome is commonly encountered in active male individuals, with a mean age diagnosis of 50 years. Having a higher prevalence in diabetes mellitus patients (247 cases per 10,000 patients per year), the incidence rate in the general population is approximately 4.3 cases per 10,000 patients per year [59,63].

### 6.2. Anatomy, Etiology, and Pathophysiology

**Figure 8:**
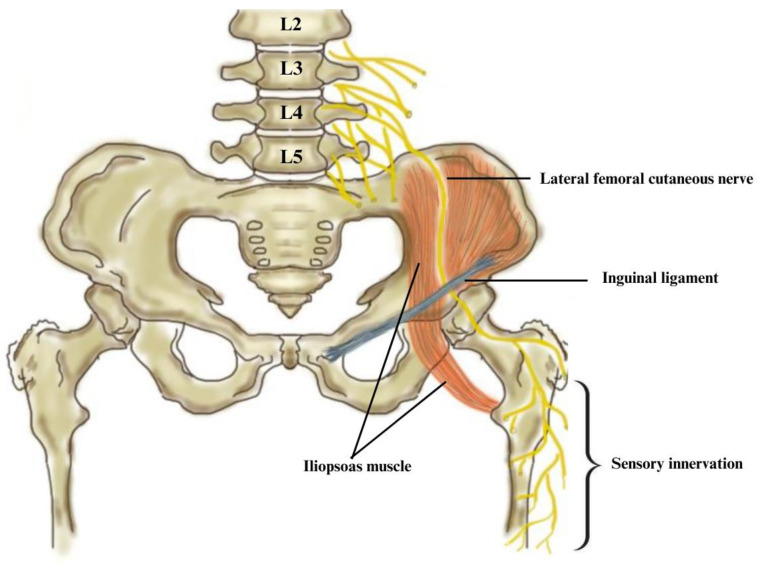
Anatomy of the lateral femoral cutaneous nerve in relation to MP. The diagram illustrates the pathway of the lateral femoral cutaneous nerve as it passes under the inguinal ligament near the anterior superior iliac spine, the area frequently involved in MP.

The LFC is a sensory nerve originating from the lumbar plexus (L1–L3). It emerges at the lateral border of the psoas major muscle and follows an oblique path from the anterior surface of the iliacus muscle to the superior iliac spine, under the inguinal ligament, anterior and medial to the sartorius muscle [59,61]. As it enters the thigh, it divides into an anterior and posterior branch [62] (Figure 8). The most common location for chronic compression or entrapment is at the exit from the pelvis. Moreover, at this level, five documented anatomical variations or exit variants are identified, which explains the variability of clinical presentation [59,61]. 

MP can be idiopathic or spontaneous, caused by: (1) mechanical factors including obesity, pregnancy, or restrictive clothing (military armor, police uniforms, jeans); and/or (2) metabolic factors such as diabetes mellitus and alcoholism [63]. However, iatrogenic cases, like post-surgical complication as a result of hip joint surgery or a prone position during spine surgery, were reported [64]. Other procedures, such as iliac bone graft extraction, appendicectomy, and caesarean section, concern isolated cases [59,61].

### 6.3. Clinical Presentation and Physical Examination

The typical presentation of patients with MP is unilateral pain, paresthesia, and numbness in the lateral or anterolateral thigh. In addition, due to high interindividual variability as a result of the nerve’s anatomical variations, burning sensation, muscle aches, or buzzing complete the clinical symptomatology [59,60]. Symptoms are usually relieved with sitting, as a result of tension reduction in the inguinal ligament, and occur or are intensified by prolonged standing or walking. Either leg seems to be affected, without preference for the dominant lower limb [58]. Motor dysfunctions are absent and deep tendon reflexes are preserved [61,62]. Furthermore, a number of provocative maneuvers such as pelvic compression, neurodynamic testing, and Tinel’s sign improve the positive diagnosis [60]. 

### 6.4. Electrodiagnostic and Imaging Techniques

LFC nerve stimulation is performed with a frequency of 1 Hz at the ASIS level, with the electrical stimulus having a duration of 0.1 ms and an intensity of 30 mA. The recording electrode is placed 14 cm distally on the oblique line created by the ASIS and patella [60]. Motor NCSs are useful to rule out other neuropathies and are usually within normal limits [61]. The EDX study is considered abnormal if the sensory nerve action potential amplitude on the affected side is 50% shorter than on the healthy side [60]. Limitations of sensory NCS include obese patients and the technical difficulties related to anatomical variation [59,60,61]. However, in obese patients where direct stimulation of the nerve is impaired, somatosensory evoked potentials (SSEP) and pain-related evoked potentials (PREP) are recommended. Nevertheless, SSEP accuracy is controversial. Some studies report a relatively low specificity of 76% and extremely low sensitivity of 52%, having a limited indication in MP diagnosis [65]. Contrary, other authors have obtained a sensitivity of 81.3%, achieving accurate findings [66]. However, both studies recommend the association of SSEP and sensory NCS for a comprehensive and accurate assessment of MP patients. PREP is a relatively new technique, having an AUC = 0.97, with a sensitivity of 91.7% and a specificity of 100% for MP diagnosis [67]. Thus, although sensory NCS studies were considered to be superior EDX methods, recent studies have concluded that modern techniques such as SSEP and PREP provide better accuracy [66,67].

US and MRN are two other important investigations which visualise the nerve trajectory, anatomical variations and morphologic changes. US is useful to evaluate the nerve and the tissue masses located in the retroperitoneal cavity. Thus, for the LFC nerve, a retrospective study yielded a cross-sectional area cut-off value of 5 mm^2^ for a positive diagnosis. However, this relatively small diameter makes it difficult to discriminate it from soft tissue [60]. Supplementarily, LFC ultrasound-guided nerve blocks can be used as a additional diagnostic tool and to differentiate from a lumbosacral radiculopathy [62]. In this technique, lidocaine is injected approximately 1 cm inferior and slightly medial to the ASIS [59,68]. MRN is also used with high accuracy in etiology, nerve injury detection, and for preoperative evaluation. Its limitations include cost burdens and lack of experience in recognizing anatomical variations [69]. 

## 7. Take-Home Messages

Lower limb entrapment neuropathies, particularly the most frequent syndromes encountered in clinical practice such as fibular nerve entrapment, proximal tibial neuropathy, tarsal tunnel syndrome, sural nerve neuropathy, deep gluteal syndrome or sciatic nerve entrapment, and lateral femoral cutaneous nerve entrapment, also known as meralgia paresthetica, are conditions that demand attention. The patients exhibit symptoms such as dysesthesia, territory-specific pain, muscle weakness, and physical signs identified through examination and pain-inducing maneuvers, which should be prioritized for suspicion over radiculopathies, plexopathies, orthopedic conditions, or gynecological pathologies in certain circumstances. These include instances when patients have held abnormal body postures (potentially during surgical procedures), carried out repetitive movements, experienced trauma, or are in postoperative stage. Professional athletes represent a high-risk demographic.

EDX studies are the primary investigative approach when nerve entrapment is suspected, which is especially applicable to the entrapment syndrome discussed in this narrative review. This analysis requires a specialist with vast experience, as entrapment neuropathies can produce ambiguous results if inadequately performed. For more complex cases where EDX results are inconclusive or if a secondary entrapment cause is suspected, implementing MRN, MRI, or ultrasound when MRN is not available, becomes crucial for confirming the diagnosis, excluding other conditions, and identifying the cause. Likewise, cross-sectional area values of nerves in the lower limb in healthy individuals have been established by a few meta-analyses. This may serve as cut-off values to distinguish between healthy nerves and entrapped nerves [70,71]. Moreover, using ultrasound elastography in symptomatic populations, nerve stiffness and longitudinal sliding of nerves in the lower limb can be evaluated for diagnosis and rehabilitation of nerve entrapment as well [72]. Additionally, ultrasound- or CT-guided injections of lidocaine at the nerve site, which can bring symptomatic relief, hold diagnostic and prognostic value.

We also believe that this manuscript can have several clinical implications. The evaluation of the peripheral nervous system is laborious and requires a good knowledge of anatomy. Also, although compression neuropathies are rare in the general population, they remain a pathology that a physician faces in the neurophysiology laboratory. Thus, a good knowledge of the symptoms and possible causes is the suitable approach for an efficient diagnosis. Moreover, the use of several diagnostics methods, such as EDX, ultrasonography, MRI, or MRN, completes the physical examination and leads to possible treatment and recovery. At the same time, with the use of modern advancements in non-invasive procedures, such as endoscopic decompression, this type of neuropathy, as described above, becomes easily manageable. Therefore, by following a systematic diagnostic approach focused on the prevalent and often complex entrapment neuropathies, treatment could be started early in the natural evolution of the disease, leading to halted nerve atrophy progression. This can result in a dramatic quality of life improvement for patients, who typically present with chronic debilitating pain in the course of the disease.

## Data Availability

Not applicable.
